# Adaptation of Autoencoder for Sparsity Reduction From Clinical Notes Representation Learning

**DOI:** 10.1109/JTEHM.2023.3241635

**Published:** 2023-02-02

**Authors:** Thanh-Dung Le, Rita Noumeir, Jerome Rambaud, Guillaume Sans, Philippe Jouvet

**Affiliations:** Biomedical Information Processing Laboratory, Ecole de Technologie SuperieureUniversity of Quebec 5623 Montreal QC H3C 1K3 Canada; Research Center at CHU Sainte-JustineUniversity of Montreal 5622 Montreal QC H3T 1J4 Canada

**Keywords:** Clinical natural language processing, cardiac failure, autoencoder, sparsity

## Abstract

When dealing with clinical text classification on a small dataset, recent studies have confirmed that a well-tuned multilayer perceptron outperforms other generative classifiers, including deep learning ones. To increase the performance of the neural network classifier, feature selection for the learning representation can effectively be used. However, most feature selection methods only estimate the degree of linear dependency between variables and select the best features based on univariate statistical tests. Furthermore, the sparsity of the feature space involved in the learning representation is ignored. Goal: Our aim is, therefore, to access an alternative approach to tackle the sparsity by compressing the clinical representation feature space, where limited French clinical notes can also be dealt with effectively. Methods: This study proposed an autoencoder learning algorithm to take advantage of sparsity reduction in clinical note representation. The motivation was to determine how to compress sparse, high-dimensional data by reducing the dimension of the clinical note representation feature space. The classification performance of the classifiers was then evaluated in the trained and compressed feature space. Results: The proposed approach provided overall performance gains of up to 3% for each test set evaluation. Finally, the classifier achieved 92% accuracy, 91% recall, 91% precision, and 91% f1-score in detecting the patient’s condition. Furthermore, the compression working mechanism and the autoencoder prediction process were demonstrated by applying the theoretic information bottleneck framework. Clinical and Translational Impact Statement— An autoencoder learning algorithm effectively tackles the problem of sparsity in the representation feature space from a small clinical narrative dataset. Significantly, it can learn the best representation of the training data because of its lossless compression capacity compared to other approaches. Consequently, its downstream classification ability can be significantly improved, which cannot be done using deep learning models.

## I. Introduction

Clinical decision support systems (CDSS) are continuously being developed and play a crucial role in promoting a personalized healthcare system, as more and more data are collected and stored continuously [1]. These data represent decisive points in advancing and enhancing the efficiency and effectiveness of CDSS operations. Predictive models have been developed based on the latter for preventive treatment and patient diagnosis, culminating in intelligent, precise, and timely healthcare improvement [2]. In one notable example, a recent study [3] analyzed the effect of CDSS on cardiovascular risk in 18,578 patients in 70 community health centers. In that case, CDSS significantly reduced the risk of cardiovascular disease among vulnerable high-risk patients.

**FIGURE 1.**
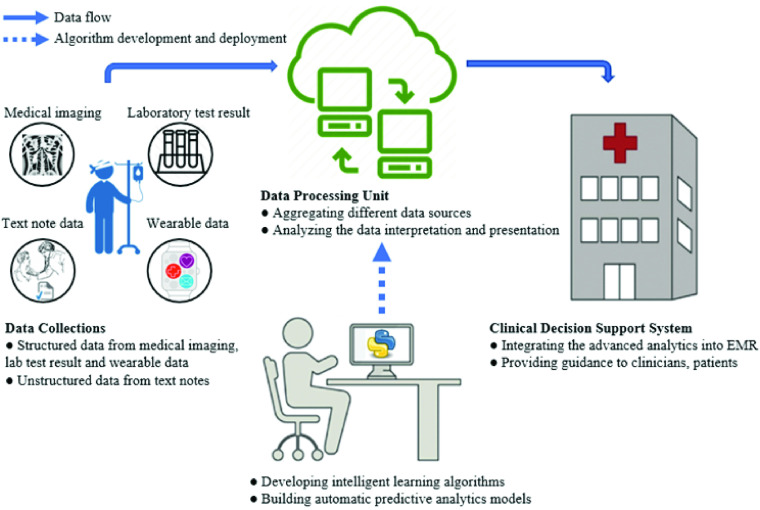
Workflow demonstration of a clinical decision-support system at CHUSJ hospital.

Following the above successes, a CDSS was developed at CHU Sainte-Justine Research Center (CHUSJ). The system monitors pediatric intensive care management for all patients ranging in age from 0 to 18 years old. Fig. 1 illustrates two fundamental processes in the CDSS workflow at CHUSJ, which involve collecting and processing critical care data. First, clinical data are collected and stored in a clinical data warehouse. The data processing unit is then systematically aggregated and processed to convert raw data to a machine-readable form in the data processing unit. This process helps analyze the unknown data interpretation and presentation. The CDSS can thus integrate the advanced analytic result of the data processing unit and learning algorithms; then, clinicians can adequately use the CDSS to guide early intervention and prevention for healthcare management.

**FIGURE 2.**
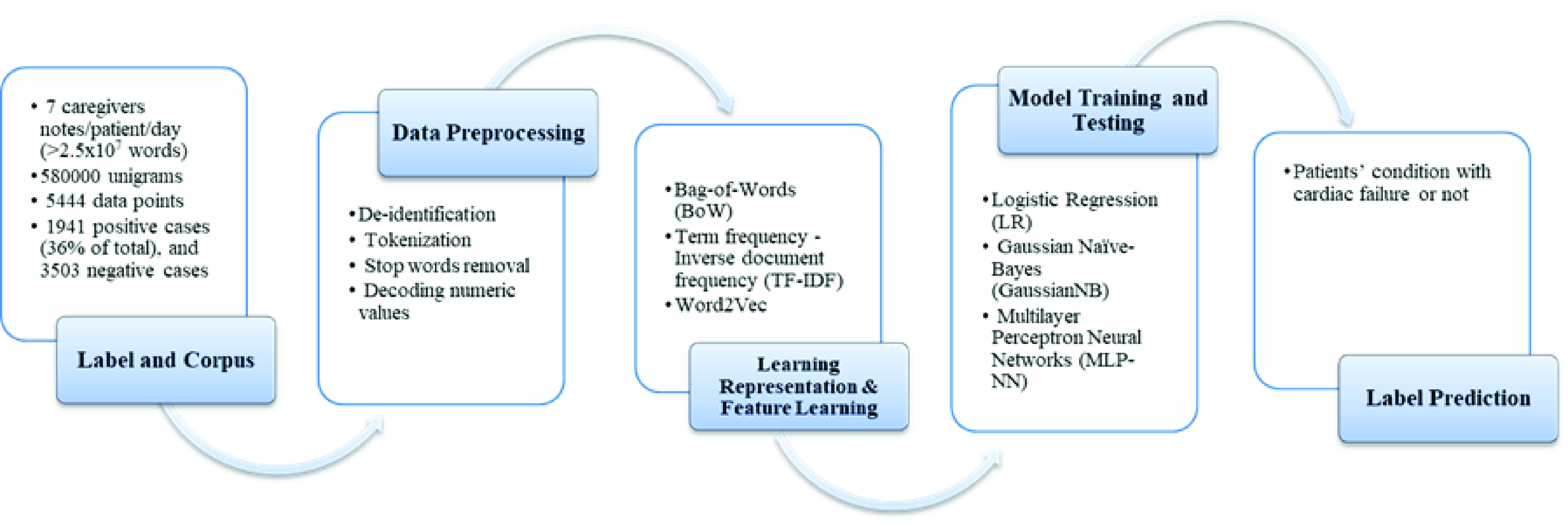
The clinical NLP based on machine learning for patients’ condition prediction at CHUSJ hospital [9].

One of the goals of the CDSS system in CHUSJ is automatically screening the data from electronic medical records, chest X-rays, and other data sources, which can increase the diagnosis rate and improve the management of acute respiratory distress syndromes (ARDS) in real time. Usually, the diagnosis of ARDS was delayed or missed in two-thirds of patients, and the diagnosis was missed completely in 40% of patients [4]. Three main conditions need to be detected to diagnose ARDS: hypoxemia (low blood oxygenation), presence of infiltrates on chest X-Ray and absence of cardiac failure [5]. Our research team has developed algorithms for hypoxemia [6], chest X-ray analysis [7], [8], and identification of the absence of cardiac failure [9], [10]. Technically, it successfully carried out extensive analyzes of machine learning algorithms (ML) aimed at detecting cardiac failure from clinical narratives using natural language processing (NLP) based on such algorithms [9]. The study’s design was to detect a cardiac failure in a patient’s first 24 hours of admission using admission notes and evolution notes within the first 24 h. As summarized in Fig. 2, the study included the clinical notes of 1386 patients classified by two independent physicians using a standardized approach. Then, a comparative analysis was performed to discover the effective combination of various representation learning techniques with different machine learning classifiers. Consequently, it confirmed that the framework proposed herein outperforms other combinations with an overall classification performance of 89% accuracy, 88% recall, and 89% precision by applying a multilayer perceptron neural network (MLP-NN) classifier in combination with a term frequency x inverse document frequency (TF-IDF) learning representation.

These results were made possible by the contributions of the feature selection process, also known as SelectKBest. The advantage of the process was proven for supervised models as the classifier performance brought overall improvements of up to 3-4% over the case without the feature selection. It is obvious to understand because there are fewer misleading features; the classifier accuracy is improved after selecting the best K features. Unfortunately, the SelectKBest feature selection continues to have certain limitations in the proposed framework. One reason is that the feature selection method is based on a statistical test that estimates the degree of linear dependency between random variables. Then, it removes irrelevant features and ignores the correlation between data elements. As a result, more samples are required for an accurate estimation and avoidance of overfitting, which is not possible in our case [11]. Furthermore, SelectKBest does not deal mainly with the sparsity of the feature space in the note representation matrix [12]. Consequently, the sparsity that characterizes the learning representation space is ignored.

In healthcare, the autoencoder algorithm (AE) has lived up to its promises and has shown its effectiveness in improving outcomes for efficient clinical decision-making. AE can find informative transformed feature vectors through the compressed latent representation. For example, a study [13] demonstrates an efficient framework for automatically learning compact representations from heterogeneous raw data sources from patient health data. In addition, AE can improve the predictability of the six different learning models to detect Parkinson’s classification [14]. Another study [15] shows that AE improved the performance of a novel outlier detection mechanism by retrofitting word vectors for the biomedical ontology matching task. In addition, having rich and accurate clinical data is very challenging [16] because the acquisition and sharing of medical data face a significant obstacle in the form of privacy issues and the sensitive nature of the data. AE can be applied for sparsity reduction in clinical representation feature to tackle problems related to limited data availability. It could effectively discover the low dimensional embeddings and reveal the underlying effective manifold structure from a sparse high dimensional document-term matrix [17].

Therefore, the present study examines alternatives to feature selection and focuses mainly on compressing data without loss of information by employing an AE algorithm. First, the study aims to achieve a better feature space without sparsity. The authors are interested in compressing the sparse TF-IDF matrix and reducing its dimensions to improve the efficiency of the feature space representation. Notably, a neural network is incorporated to learn efficient codings of unlabeled data to address the issues caused by sparse vectors generated from the TF-IDF representation feature space for clinical notes. Then, the compressed vector space from the TF-IDF matrix is fed into the classifiers as a refined input. Finally, ML classifiers conduct the learning process to draw comparative results, which are then used to evaluate the classification performance.

Our study confirms that AE effectively compresses the vector space of the TF-IDF representation for clinical narratives into a lower dimension. The proposed approach can retain the critical feature by capturing the correlation between attributes during the training process, hence; the downstream classification task can generally be increased to 2-3% for each evaluation criterion. Furthermore, the value of AE behaviors in a limited data set is also highlighted. The working mechanism of the AE is analyzed and explained how the AE works to compress data through the encoder and decoder. Based on the information-theoretic framework, the working mechanism of the AE is to optimize the information bottleneck during the compression and prediction process, respectively. As a result, the behavior of AE in limited data is exactly in harmony with such cases where there is much larger data availability. Section II will discuss the materials and methods. The experimental results and discussion then will be discussed in section III, IV. Finally, section V provides concluding remarks.

## II. Materials and Methods

### A. Data Sparsity Challenges

In numerical analysis, a sparse matrix or array is a matrix in which most elements are zero [18]. The number of zero-valued elements divided by the total number of elements (e.g., $m \times n$ for a $m \times n$ matrix) is called the matrix sparsity (equal to 1 minus the density of the matrix). Using these definitions, a matrix will be sparse when its sparsity is more significant than 0.5. In our case, after the research ethics board approved the research protocol from the Research Center of the Sainte-Justine Hospital, the data were retrospectively extracted from the electronic medical record. There are more than 580000 (unigrams) word count from 5444 single lines of notes with 1941 positive cases (36% of total) and 3503 negative cases. All the notes are short narratives, and detailed description characteristics can be found in the Supplementary Materials from [9]. The longest n-gram is over 400 words, but most n-gram length distribution is between 50 and 125 words. The average length of the number of characters is 601 and 704. And the average size of the number of digits is 25 and 26 for the positive and negative cases, respectively. Then, the data was pre-processed by applying the stop-word removal to exclude the minor information. In addition, the negation in medical expression was used to add the negative meaning from French notes. For the vital numeric values (heart rate, blood pressure, etc.), all numeric values for vital sign values were kept (nearly 4% of the notes), and the decoding for those number values was used to decode the numeric values. Finally, the feature selection, SelectKBest, was used to select the top best ‘k=20000’ of the vectorized features for the TF-IDF representation learning feature space. Hence, there is a matrix of features of $(5444 \times 20000)$. It is calculated by the Eq. 1, and the sparsity of the matrix is greater than 0.9.

It confirms that the representation matrix from the TF-IDF is sparse because every word is treated separately. Hence, the semantic relationship between separated entities is ignored, which would cause information loss. Although the combination of TF-IDF and MLP-NN consistently outperformed other combinations with overall performance and was the most stable under all circumstances [9], the sparsity remains. Therefore, the motivation is to compress the sparse, high-dimensional data by reducing the dimension from the TF-IDF feature space of clinical notes representation

\begin{equation*} \text {sparsity}= 1 - \frac {\text {count}\_ \text{nonzero(TF-IDF)}}{\text {total}\_ \text{elements}\_ \text{of}\_ \text{(TF-IDF)}} \tag{1}\end{equation*}

### B. Autoencoder Learning Algorithm

An AE was originated by [19] to solve a nonlinear dimensional reduction; later, AE was famously promoted by training an MLP-NN with a small central layer to reconstruct high-dimensional input vectors [20], [21]. Technically, AE takes an input $X \in \mathcal {R}^{N \times D}$ and maps it to a latent representation $Z \in \mathcal {R}^{N \times M}$ via a nonlinear mapping. Let us call $x \in X$, and $z \in Z$, then it will be as:

\begin{equation*} z=g(Wx+b) \tag{2}\end{equation*}

$W$ is a weight matrix during training, $b$ is a bias vector, and $g(\cdot)$ stands for a nonlinear function, such as the logistic sigmoid function or a hyperbolic tangent function. The encoded feature representation $x$ is then used to reconstruct the input $x$ by reverse mapping, leading to the reconstructed input $x'$:

\begin{equation*} x' = f(W'z+b') \tag{3}\end{equation*}

where $W'$ is usually limited to the form of $W'=W^{T}$, i.e., the same weight is used to encode the input and decode the latent representation. $f(\cdot)$ is also a non-linear function. The AE tries to learn a function $f_{W', b'}(x) \approx x'$. In other words, it is trying to learn an approximation of the identity function for the output $x'$ that is similar to $x$. Still, by placing constraints on the network, such as limiting the number of hidden units, interesting data structures can be discovered. Then, the reconstruction error is defined as the Euclidean distance between $x$ and $x'$ that is constrained to approximate the input data $x$ (that is, minimizing $||x-x'||^{2}$).

\begin{align*} \mathcal {L}\left ({x, x'}\right)=&\left \|{x-x'}\right \|^{2} \\=&\left \|{x-f(W'\left ({g(Wx+b) }\right)+b') }\right \|^{2} \tag{4}\end{align*}

For the reconstruction evaluation between the original data $x$, and the reconstructed output $x'$, the statistical measure $R^{2}_{i}$ will be applied for the $i^{th}$ variable of $x_{i}$, and it can be computed as:

\begin{equation*} R^{2}_{i} = 1 - \frac {\sum _{j=1}^{m} (x_{j,i} - x'_{j,i})^{2}}{\sum _{j=1}^{m} x_{j,i}^{2}} \tag{5}\end{equation*}

Since $R^{2}=1$ will be a perfect reconstruction. Consequently, the reconstruction will be evaluated by how much the value of $R^{2}$ is close to 1.

Ideally, an effective AE can be designed and trained based on the minimization of reconstruction error from Eq. 4 and maximization of the reconstructed effectiveness from Eq. 5; however, it is substantially based on its width (number of neuron units or latent representation dimension $M$) and its depth (number of hidden layers). First, conventional AE relies on the dimension of the latent representation $z$ being smaller than that of the input $x$ ( $M < D$), which means that it tends to learn a low-dimensional compressed representation. The study [22] presents methods to learn the decoder function $f(\cdot)$ as a learnable function through the reconstruction error in Eq. 4 in several representation learning approaches. It is concluded that the compression depends on dimension $M$ but less on dimension $D$. Second, it has been shown that training a neural network-based by increasing the number of hidden layers (in combination with an increase in the number of neuron units per layer) achieves less consistent results [23]. Therefore, a small and simple AE will be used in our case. An AE with three layers (one input layer, one hidden layer, and one output layer) is employed. Mainly, to reduce the parameters from the latent space of the AE, the regularization technique is applied from study [24] to remove redundant parameters.

**FIGURE 3.**
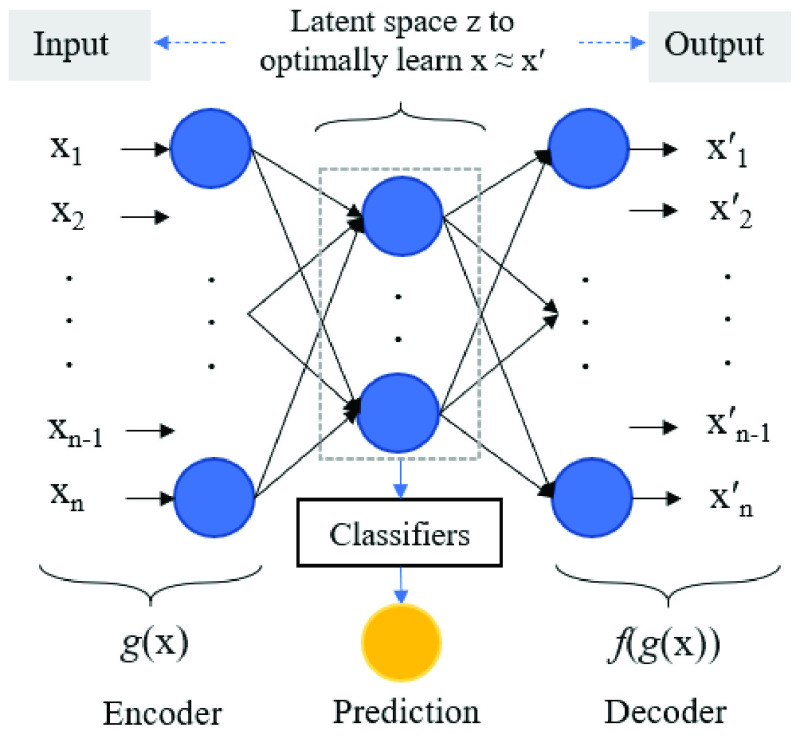
Schematic structure of an AE-based for compression and prediction.

After training, the weight matrix from the hidden layer as a pre-trained tool is used. A classifier subsequently uses this pre-train latent space representation to perform the binary classification, as shown in Fig. 3. For the classifiers, it is essential to have consistency in evaluating the proposed approach’s performance. Then, six different ML classifiers, including Random Forest (RF), Multinomial Naive Bayes (MultinomialNB), Logistic Regression (LR), Support Vector Machine (SVC), Gaussian Naive Bayes (GaussianNB), and Multilayer Perceptron Neural Network (MLP-NN) are used.

Furthermore, to understand the dynamics of learning and the behavior of AE, particularly in our case with limited data, the behavior of AE during the training process from the encoder and decoder is analyzed. Technically, it is captured to understand how the AE can retain the information during the compression process. To do that, the information-theoretic quantities and their estimators are applied. The technique is based on information-theoretic learning, which computes and optimizes information-theoretic descriptors named mutual information. The information-theoretic framework [25], [26], [27] has been utilized for a detailed theoretical explanation of an AE. These studies rely on the “information bottleneck” [28], [29] to understand and estimate how the AE works by quantifying its information plane coordinates. The information bottleneck can be used as an optimal bound that maximally compresses the input $x$, for a given mutual information on the desired output $x'$. There are comprehensive overviews of recent studies [30], [31], [32]. Technically, the output activation is firstly binned as stated in [29], and each hidden layer $i$ ( $1 \leq i \leq K $) is treated as a single variable $T_{i}$. Then it will be able to estimate the mutual information between all the hidden layers and the input/output layers by estimating the joint distribution $P(X,T_{i})$ and $P(T_{i},X')$, and use them to calculate the mutual information of the encoder (between the input $X$ and the hidden layer $T_{i}$), and the mutual information of the decoder (between the hidden layer $T_{i}$ and the desired output $X'$) using the following equations Eq. 6, 7. Finally, the good representation $T(X)$ can be learned, which is characterized by its encoder and decoder distribution $P(T|X)$, and $P(X'|T)$, respectively, to effectively map the input patterns $X$ to a good prediction of the desired output $X'$.

\begin{align*} I(X;T_{i})=&\sum _{x\in X, t\in T_{i}}P(x,t)\log \left({\frac {P(x,t)}{P(x)P(t)}}\right) \tag{6}\\ I(T_{i};X')=&\sum _{t\in T_{i}, x'\in X'}P(t,x')\log \left({\frac {P(t,x')}{P(t)P(x')}}\right). \tag{7}\end{align*}

## III. Experimental Implementation

To assess the performance of our method, metrics including accuracy, precision, recall (or sensitivity), and F1 score were used [33]. These metrics are defined as follows.

\begin{align*} \text {Accuracy (acc) }=&\frac {\mathrm {TP}+\mathrm {TN}}{\mathrm {TP}+\mathrm {TN}+\mathrm {FP}+\mathrm {FN}} \\ \text {Precision (pre) }=&\frac {\mathrm {TP}}{\mathrm {TP}+\mathrm {FP}} \\ \text {Recall/Sensitivity (rec)}=&\frac {\mathrm {TP}}{\mathrm {TP}+\mathrm {FN}} \\ \text {F1-Score (f1)}=&\frac {2^{\star } \text {Precision}^{\star } \text {Recall}}{\text {Precision }+\text {Recall}}\end{align*}

where TN and TP stand for true negative and true positive, respectively, and are the number of negative and positive patients correctly classified. FP and FN represent false positives and false negatives, respectively, and represent the number of positive and negative patients incorrectly predicted.

For implementation, the same hyperparameters are used as from the previous study [9] for all classifiers to have a consistent evaluation of the performance: avoiding overfitting by applying the dropout (p=0.25) [34], and the GlorotNormal initializer [35]; balancing the classes by using the Bayes Imbalance Impact Index [36] to deal with the imbalanced classes. The data was also divided into 60% training, 20% validation, and 20% testing. The implementation was done using Python Scikit learn [37] and Keras [38].

There is a tradeoff between the guarantee to identify the best combination of hyper-parameters and the computation time. And, for training a neural network, usually, only some hyper-parameters matter. The others have little impact on the machine learning model’s accuracy. Based on the study [39], there are three essential hyper-parameters, including the number of hidden layers, the number of nodes on each hidden layer, and the learning rate for the backpropagation algorithm. With this limited range of hyper-parameters, the grid search will quickly become feasible to optimize every parameter simultaneously, including the cross-product of all intervals. Then, the models can be trained quickly. Further advantages of grid search include easier parallelization and flexible resource; the equivalent does not hold for Bayesian optimization [40]. Therefore, this study used grid search for up to three hidden layers and 500 neurons per layer, and other hyperparameters are summarized in Table 1 for AE training. For the optimizers, the Stochastic Gradient Descent (SGD) and Adaptive Moment Estimation (ADAM) was used with small scalar $\epsilon $, and the forgetting factors for gradients and second moments of gradients, $\beta _{1}$ and $\beta _{2}$. Then, a combination with the highest estimations was considered the best performance.

**TABLE 1:** Hyperparameters Summary for AE Trainning

Hyperparameter	Ranges
Hidden layers	1–3
Neurons	100–500
Activation	Sigmoid
Kernel initializer	GlorotNormal
Optimizers	SGD, ADAM
Learning rate	0.001 - 0.01
$\beta _{1}$	0.9
$\beta _{2}$	0.999
$\epsilon $	$e^{-8}$- $e^{-7}$

## IV. Results and Discussion

To deal with sparsity, many researchers focus on dimension reduction. There are two most popular techniques, namely Linear Discriminant Analysis (LDA) and Principal Component Analysis (PCA), for their simplicity among other dimension reduction techniques [41], even with a large dataset [42]. Especially when the training data set is small, and the PCA-supervised discriminative approach can outperform, it is also less sensitive to the variability of the training sets [43]. The study [44] shows that PCA can increase the performance of different ML classifiers for predicting cardiac failure.

It can be said that the classifiers performed better after applying LDA to the linear data set. If the classes are non-linearly separable, the LDA cannot effectively discriminate between these classes [45]. Otherwise, in the case of linear data, LDA can reduce the dimensionality and be used in different classification tasks [46]. However, the TF-IDF enhanced with the LDA approach did not allow the classifier to score high accuracy compared to the other two methods when smaller datasets were fed [47]. One of the reasons was explained in [42]; the results showed that ML algorithms with PCA produce better results when the dimensionality of the data sets is high. When the dimensionality of datasets is low, the ML algorithms without dimensionality reduction yield better results. Another possible way is using an unsupervised generative Latent Dirichlet allocation to estimate the topic distribution (topics) by using observed variables (words). Latent Dirichlet allocation shows the effectiveness of overcoming the sparsity from the feature space matrix of TF-IDF [48]. It can also help to make texts more semantically focused and reduce sparseness [49]. However, its selection of characteristics does not improve performance with small data [50].

**FIGURE 4.**
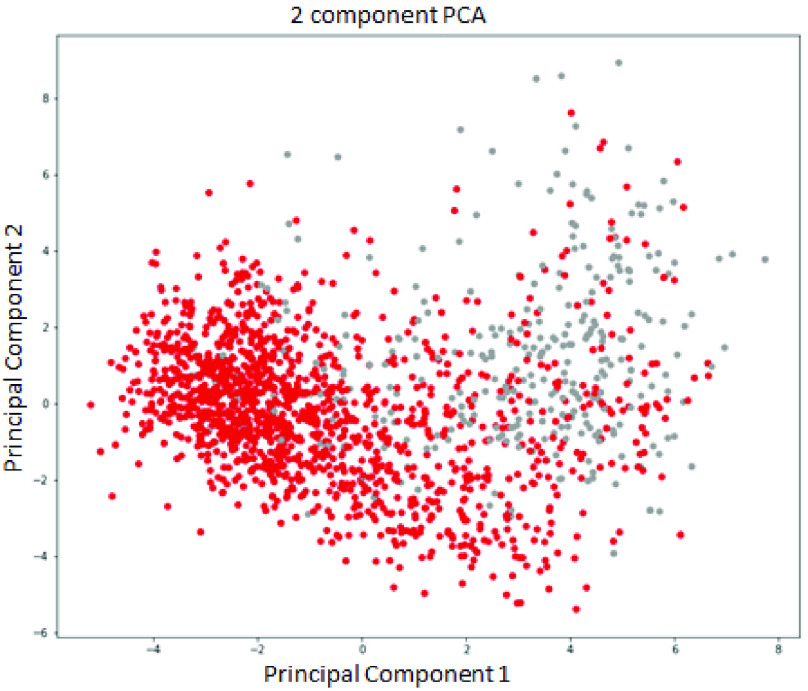
Visualization of the representation space for 2 components from Principle Component Analysis (PCA).

**FIGURE 5.**
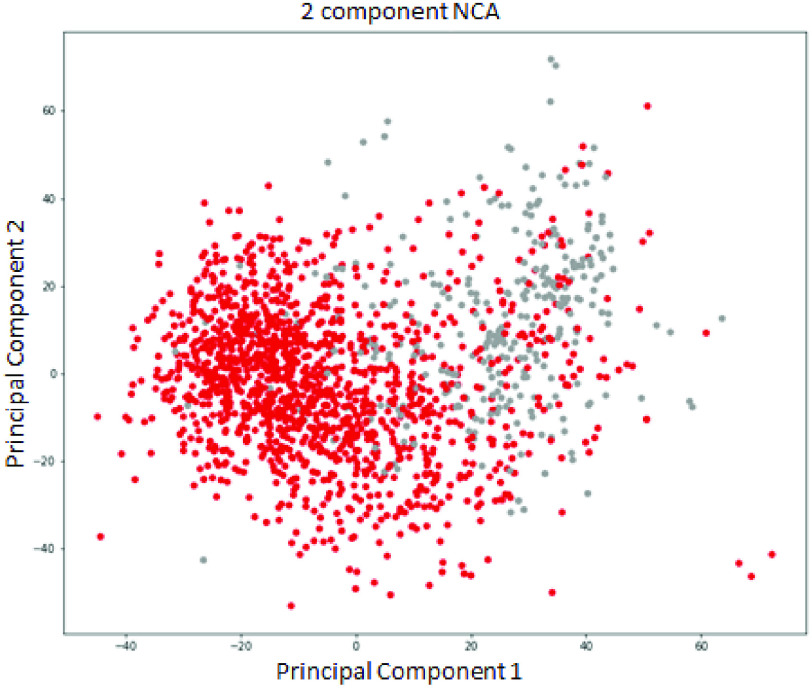
Visualization of the representation space for 2 components from Neighborhood Component Analysis (NCA).

The possibility of PCA for sparsity reduction was explored because of the advantages mentioned above. The training was tuned and performed, and the best performance was achieved by decreasing to 2 principal dimensions. The completed test has an accuracy of 88%, a precision of 88%, a recall of 86%, and an f1-score of 87%. Furthermore, following the recommendation of [51], a statistical method, Neighborhood Component Analysis (NCA) [52], was also used to reduce the dimensions of the data set. NCA has shown that it works well on a small dataset for the medical domain. However, the result is slightly better than PCA; NCA only achieves an accuracy of 89%, a precision of 88%, a recall of 89%, and an f1-score of 88%. From Fig. 4, 5, it can be easily seen the features overlap; hence, the classification task hardly separates the boundary for the binary classification. Neither PCA nor NCA can improve classification performance summarized in Table. 2. It confirms the limitation of these approaches by linearly approximating a feature subspace to maximize class separability.

**TABLE 2:** A Comparison Performance of Feature Selection Approaches

Feature selection	Accuracy	Precision	Recall	F1
SelectKBest [9]	0.89	0.89	0.88	0.88
PCA	0.88	0.88	0.86	0.87
NCA	0.89	0.88	0.89	0.88

Furthermore, the non-linear activation function AE performs best on compression of the sparse TF-IDF representation space. This study compares the effectiveness of reconstruction based on the reconstruction evaluation from Eq. 5 between PCA, linear activation function AE (LAE), AE, and stacked AE (SAE) [53]. The results confirm that the PCA and LAE have the same performance, achieving about 80% of the reconstruction. When the activation of AE is linear, then PCA and LAE are identical. There is no improvement if the SAE is used to extract the features in cases of limited data. Besides, the effectiveness of non-linear activation in AE is proved when it can maximally reconstruct up to 86% compared to the original spare data. It is one of the advantages of nonlinear transformation from AE, trained by a neural network, which is superior to the linear transformation from other approaches.

**FIGURE 6.**
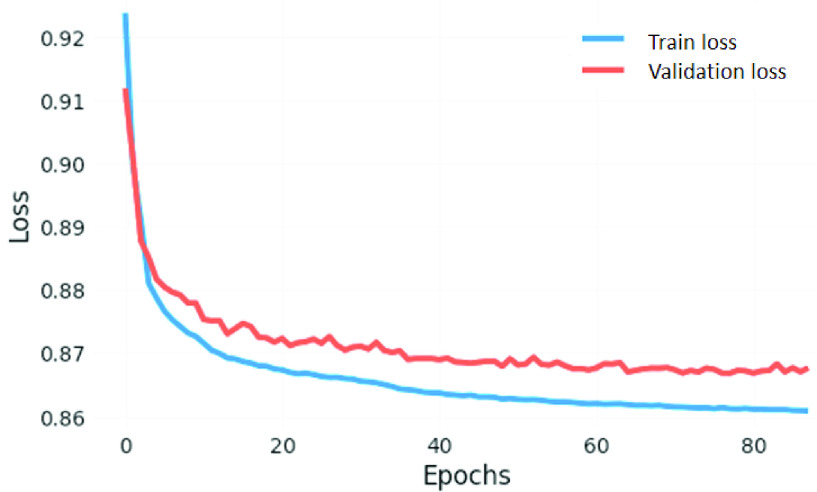
Loss for training and validation for the AE algorithm.

**FIGURE 7.**
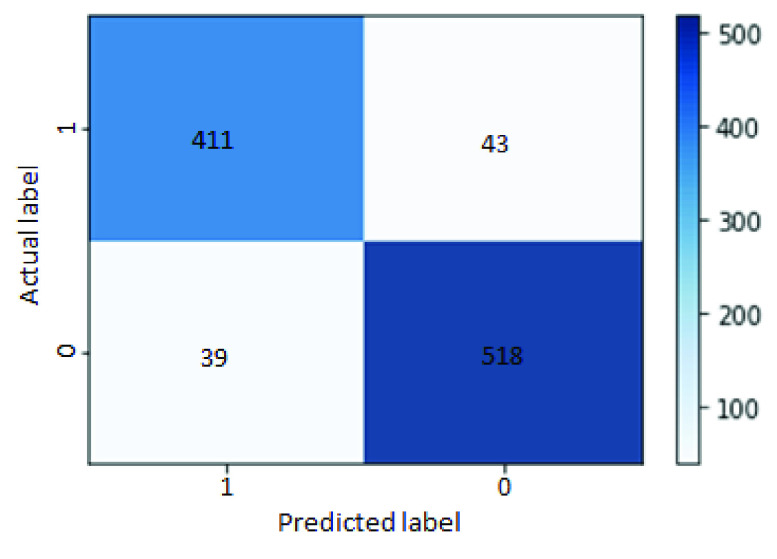
Confusion matrix of the MLP-NN classifier, showing the classification of positive (1) and negative (0) between predicted and actual labels.

Overall, the downstream classification performances are effectively improved by feeding the compressed feature space output from the AE to ML classifiers. Fig. 6 shows the loss during the training and validation process by optimizing the loss function from Eq. 4 for training the AE; both training and validation losses have quite-smooth convergence. After successfully training the AE, there is a pre-trained compressed, low-dimension feature space. Then, machine learning classifiers are employed to perform the classification and evaluate the performance. Instead of performing on MLP-NN, LR, and GaussianNB, it is also tested with other classifiers such as Random Forest (RF), Multinomial Naive Bayes, and Support Vector Machine. The best performance from MLP-NN classifier is achieved at 92%, 91%, 91%, and 91%, respectively, for accuracy, precision, recall, and f1 score. And the detailed confusion matrix showing the classification of positive cases (1) and negative cases (0) between predicted and actual labels for the holdout set is shown in Fig. 7. The experimental results are improved to 2–3 % for each evaluation criterion from [9], which had a general classification performance in a sparse TF-IDF feature space at 89% accuracy, 89% precision, 88% recall, and 88% f1 score. It confirms that the AE method can deal with sparsity by compressing the TF-IDF feature space. Consequently, it improves the downstream task performance of the MLP-NN classifier and is more robust than other methods. Recent work [54] also confirmed a similar effect, but it was applied to a different dataset type and larger data availability. These results confirm the effectiveness of compressing the feature representation learning space into a low-dimensional representation using the AE algorithm. The robust transformation can outplay the deep learning models with limited data resources.

**FIGURE 8.**
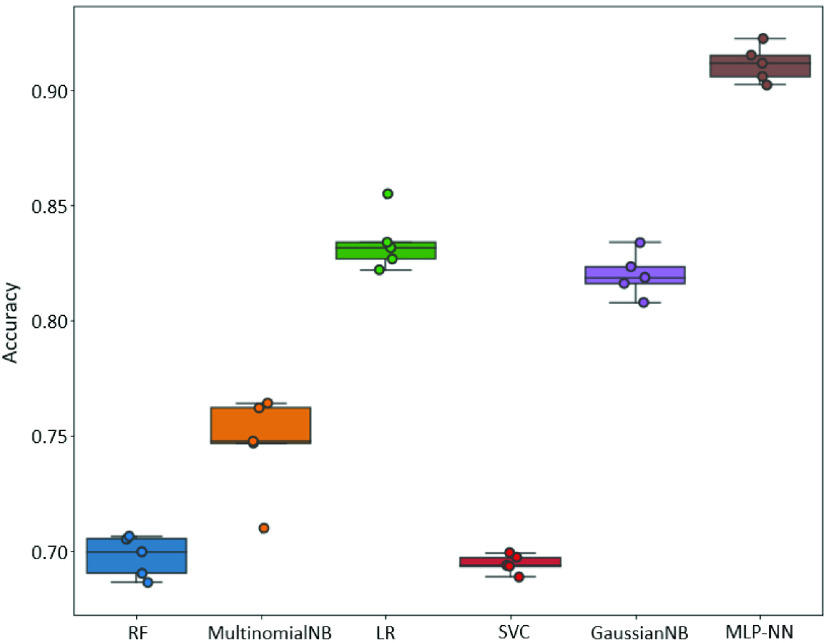
A comparison evaluation of the box plot 5-fold cross-validation results for classifiers performance.

Cross-validation was further used to accurately estimate the model’s predictive performance and determine the reliability of ML algorithms [55]. Fig. 8 shows the accuracy comparison, using a box plot, of the 5-fold cross-validation. It can be seen that the best three classifiers are MLP-NN, LR, and GaussianNB, respectively. All their median accuracy is over 80%; mainly, the MLP-NN classifier’s median accuracy is the highest, over 90%. While there is not much difference between LR and GaussianNB, the median accuracy is around 82-83%. In addition, MultinominalNB, RF, and SVC follow right after as the three most minor performances, respectively, with median accuracy lower than 75%. Second, although the models’ performance is assumed that the returns of accuracy follow a normal distribution, in reality, the returns are usually skewed. Notably, there is two skewness of the accuracy distribution for all classifiers. There is a negatively skewed distribution (skewed left) from the MLP-NN, LR, and RF, which may expect frequent smaller accuracy than their median in practice. In contrast, it should be expected to have higher accuracy than the median from the GaussianNB, MultinominalNB, and SVC because they all have positively skewed distribution (skewed right). Lastly, the dispersion distribution for most classifiers’ accuracy is quite similar because the variability range contains all the smallest and largest accuracy values at the end of the whiskers. However, there is an exception for the LR and MultinomialNB classifiers, which have values outside the box plot’s whiskers. It means that the two classifiers are less stable and reliable. In short, MLP-NN gives the best performances because of its high and stable accuracy for the model generalization validation; GaussianNB follows right after; LR is comparatively similar to GaussianNB. And all other classifiers are less effective.

**FIGURE 9.**
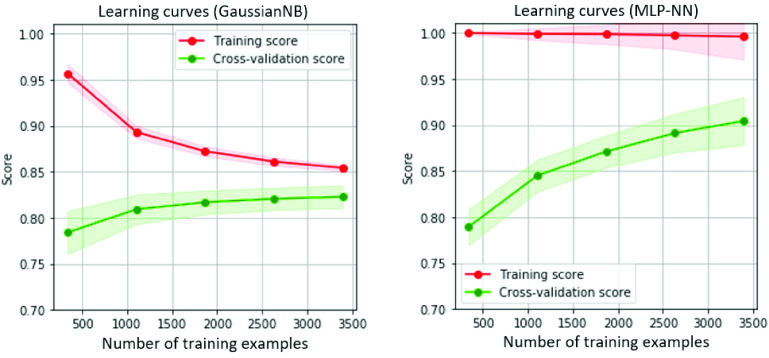
Performance of classifiers in case of increasing the training size: GaussianNB (left) and MLP-NN (right).

Furthermore, an important aspect of performance analysis is that the proposed approach still shows its advantageous capacity to increase data availability. The study investigated the effectiveness of AE for compressing feature space and studied how algorithm performance varies with the increasing of training examples from the compressed feature space. The performance of two classifiers, GaussianNB and MLP-NN, was assessed to evaluate their effectiveness. When it possibly increases data availability in the future, whether the classifier improves performance or not. In this case, study [56] confirms that when the number of training examples increases, the generative model based on Naive Bayes would expect to perform better. However, our results are in contrast to that confirmation. Fig. 9 shows the GaussianNB (left) and MLP-NN (right) training and validation scores when increasing the number of training examples. Technically, the GaussianNB reaches a plateau of performance after around the $2000^{th}$ training examples with the same dataset size, and the cross-validation score could not improve. It should be expected that this is one of the limitations of GaussianNB, namely the linear discrimination characteristic for a real-world dataset, discussed in [57]. In contrast, the MLP-NN shows improvement with the increasing size of the dataset. Its cross-validation score gradually increases from the point at $500^{th}$ to the $2500^{th}$ training examples; especially, the slope shows no signs of decreasing after reaching the maximum number of the training example. In short, GaussianNB shows improvement, but not as much as the MLP-NN, and reaches a plateau more quickly. It can be confirmed that our approach with MLP-NN is still applicable when data is possibly increased and continually improves its classification performance.

**FIGURE 10.**
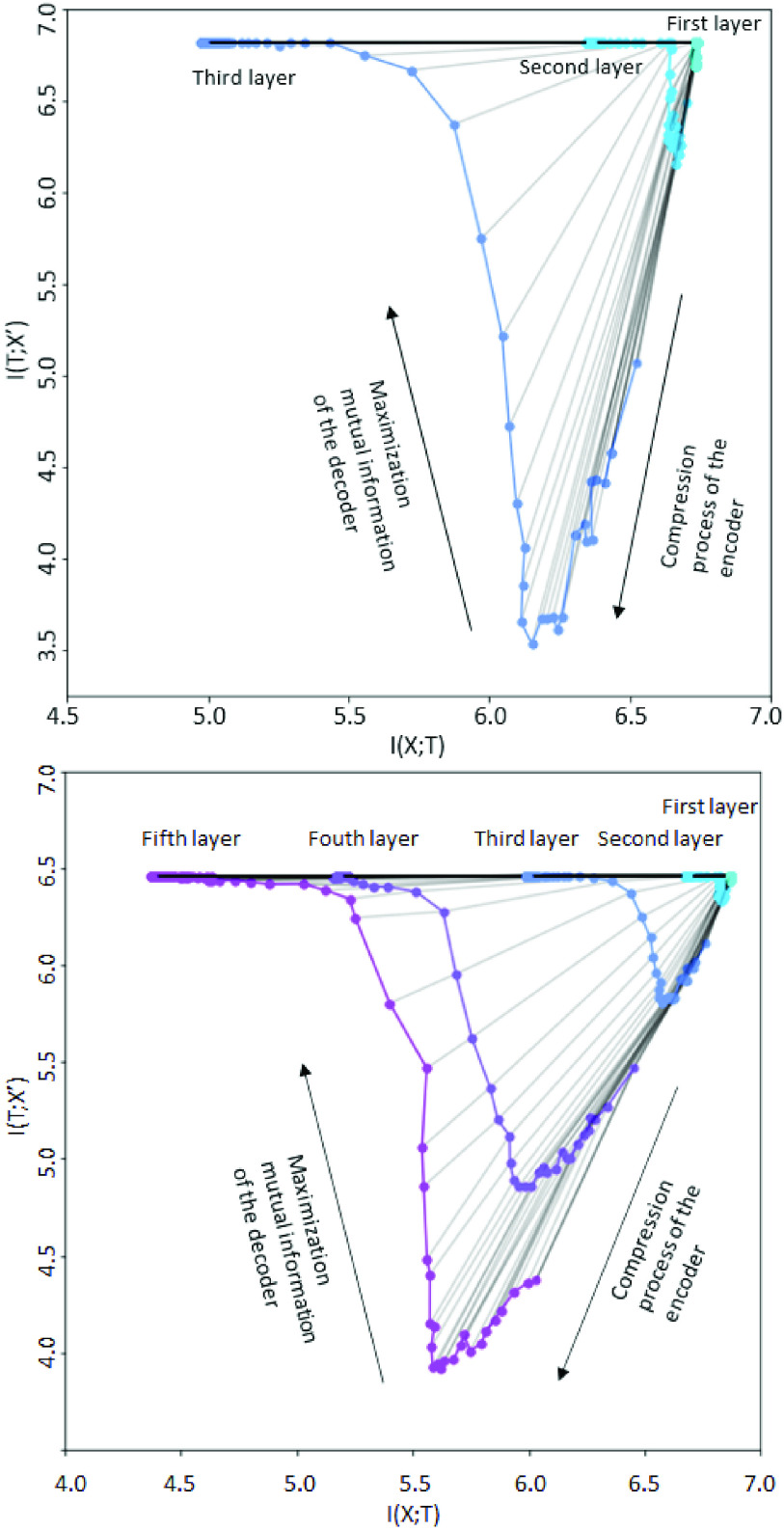
The evolution of the layers with epochs in the information plane for three hidden layers (top) and five hidden layers (bottom).

Moreover, the behavior of AE in limited data is in harmony with more significant data cases based on the information-theoretic framework. The behavior of AE was analyzed, and the technique was based on an information-theoretic framework, as mentioned in Eq. 6, and 7. It aims at understanding how the AE behaves during the compression process by analyzing the mutual information of each hidden layer from the encoder and decoder. Generally, this type of analysis has been performed for a larger data set and has mainly focused on other data sources compared to our case; such as computer vision [58], medical imaging [59], and genetics [60]. The analysis for two AE models was performed concerning various hidden layers (three hidden layers and five hidden layers). As shown in Fig. 10, there are two phases of the information plane in each hidden layer of the three-layer and five-layer cases. It is noted that from left to right, it illustrates the behavior of each hidden layer. And in each hidden layer, from top to bottom, it captures the mutual information for each training epoch. Finally, all trajectories follow a similar path during the learning process, eventually converging and getting closer to the optimal points in the bottleneck bound.

Specifically, it can be divided into two phases for the working mechanism of AE in Fig. 10. The first phase is called the drift phase, where the AE attempts to learn the latent representation $T(X)$ with a smaller dimension than the original data $X$. During the compression, there will be information loss, which is why it can be seen the trend of decreasing the mutual information of encoder $I(X; T)$. At the end of this step, there will be a compressed latent representation $T(X)$, and optimal mutual information $I(X; T)$. Then, the second phase is named the diffusion phase. Within this step, the AE tries to find the reconstructed data $X'$, which is optimally close to the original data $X$. The AE maps the latent representation $T(X)$ to the reconstructed data $X'$ by maximizing the mutual information of the decoder $I(T; X')$. By doing that, there is an increasing trend of $I(T; X')$; until $I(T; X')$ reaches its optimal bound for each layer. And the optimal mutual information will get smaller when AE has more hidden layers. In the case of three hidden layers, the optimal mutual information of the encoder $I(X, T)$ is larger by 6.0 but is maximum at 5.5 for five hidden layers. It is the same for the optimal mutual information of the decoder $I(T, X')$ at nearly 7.0 and 6.5 for three and five hidden layers, respectively. These results illustrate the mechanism of an AE is to optimize the information bottleneck trade-off $T(X)$ during compression and prediction for each layer. Remarkably, it is trained on a small and sparse dataset; still, it proves its effectiveness by compressing and maximizing the mutual information from the TF-IDF feature space.

## V. Conclusion

First, this study has shown that the participation of an AE in training can effectively compress the feature space of TF-IDF. The AE with a nonlinear activation function can achieve the reconstruction capacity at 86% compared to the original data. It outperforms other approaches such as PCA, NCA, LAE (AE with linear activation function), and stacked AE. It concludes that AE can learn the best representation of the training data due to its lossless compression capacity.

Additionally, the AE also works well with a small clinical dataset, especially in harmony with the information-theoretic mechanism of an AE for a larger dataset and from different data sources. It has two learning phases; the encoder’s drift phase by trying to compress the data. The second phase is related to the diffusion phase by maximizing the mutual information process in the decoder. Consequently, it shows the effectiveness of lost information in compressing the data. By doing so, the interpretability can also be captured as comprehensibility and transparency of the proposed model for decision-making in our CDSS system recommended by [61].

The second step involves using an MLP-NN to predict the health status based on the compressed feature space. It has been shown that the sparsity reduction for the feature space strongly affects the classifier performance in the downstream task. AE learning algorithm effectively leverages the sparsity reduction. As a result, it helps the MLP-NN classifier achieve 92% accuracy, 91% recall, 91% precision, and 91% f1-score. This efficient ensemble model can outperform all alternative approaches: GaussianNB, LR, RF, MultimonialNB, and SVC.

The proposed approach is still proving successful in cases where data availability is increased. The MLP-NN effectively achieves a better performance after the GaussianNB reaches its maximum capacity. In future work, the optimal parameters will be chosen, and our method will be validated on more datasets. The weak supervision approach will be explored, as it recently proved its effectiveness in 4,000 cardiac magnetic resonance sequences with imperfect labels [62]; because it can maximize unlabeled data at scale, which is costly to annotate.

Finally, the CDSS is still under development. By combining this NLP algorithm to detect the absence of heart failure with the two other algorithms already developed on hypoxemia detection [6] and chest X-ray analysis [7], [8], the next step of our study is to implement the resulting CDSS (integration of the three algorithms) within the cyberinfrastructure of the pediatric intensive care unit (PICU) at Sainte-Justine Hospital to diagnose ARDS early. We will then verify the ability of the CDSS to detect ARDS prospectively once the integration with the PICU e-Medical infrastructure will be completed.
